# Evolutionary History of LINE-1 in the Major Clades of Placental Mammals

**DOI:** 10.1371/journal.pone.0000158

**Published:** 2007-01-17

**Authors:** Paul D. Waters, Gauthier Dobigny, Peter J. Waddell, Terence J. Robinson

**Affiliations:** 1 Evolutionary Genomics Group, Department of Botany and Zoology, University of Stellenbosch, Matieland, South Africa; 2 Comparative Genomics Group, Research School of Biological Sciences, The Australian National University, Canberra, Australia; 3 Institut de Recherche pour le Développement, Centre de Biologie pour la Gestion des Populations, Montferrier-sur-Lez, France; 4 Laboratory of Biometry and Bioinformatics, University of Tokyo, Tokyo, Japan; 5 South Carolina Cancer Center, University of South Carolina, Columbia, South Carolina, United States of America; University of Queensland, Australia

## Abstract

**Background:**

LINE-1 constitutes an important component of mammalian genomes. It has a dynamic evolutionary history characterized by the rise, fall and replacement of subfamilies. Most data concerning LINE-1 biology and evolution are derived from the human and mouse genomes and are often assumed to hold for all placentals.

**Methodology:**

To examine LINE-1 relationships, sequences from the 3′ region of the reverse transcriptase from 21 species (representing 13 orders across Afrotheria, Xenarthra, Supraprimates and Laurasiatheria) were obtained from whole genome sequence assemblies, or by PCR with degenerate primers. These sequences were aligned and analysed.

**Principal Findings:**

Our analysis reflects accepted placental relationships suggesting mostly lineage-specific LINE-1 families. The data provide clear support for several clades including Glires, Supraprimates, Laurasiatheria, Boreoeutheria, Xenarthra and Afrotheria. Within the afrotherian LINE-1 (AfroLINE) clade, our tree supports Paenungulata, Afroinsectivora and Afroinsectiphillia. Xenarthran LINE-1 (XenaLINE) falls sister to AfroLINE, providing some support for the Atlantogenata (Xenarthra+Afrotheria) hypothesis.

**Significance:**

LINEs and SINEs make up approximately half of all placental genomes, so understanding their dynamics is an essential aspect of comparative genomics. Importantly, a tree of LINE-1 offers a different view of the root, as long edges (branches) such as that to marsupials are shortened and/or broken up. Additionally, a robust phylogeny of diverse LINE-1 is essential in testing that site-specific LINE-1 insertions, often regarded as homoplasy-free phylogenetic markers, are indeed unique and not convergent.

## Introduction

The non-LTR retrotransposons Long Interspersed Nuclear Elements-1 (LINE-1, or L1) are a major component of mammalian genomes (∼20% of that of human) that transpose through an RNA intermediate (reviewed in [1]). Most LINE-1 elements are 5′ truncated upon transposition and are therefore inactive. In fact, only an estimated 60 LINE-1 copies are potentially active in the human genome [2]. LINE-1 have been shown to be responsible for many genetic disorders such as gene disruption, nucleotide deletions, duplications and chromosomal instability through heterologous recombination ([3] and references therein). However, they are also involved in important genomic functions. These include regulation of gene expression [4], [5] and possibly X-inactivation in females [6], [7]. Further, LINE-1 provides the Reverse-Transcriptase (RTase) necessary for transposition of the ALU SINE sequences [8], and may also have a role in the generation of processed pseudogenes [9].

Most of the current data concerning LINE-1 biology and evolution result from investigations of the human and mouse genomes and are often assumed to hold for all eutherians [1]. Previous studies have investigated the paleohistory of LINE-1 families based on the “genomic fossil record of pseudogenes retroposed at different times from active source genes” [10]. Unfortunately this usually relies heavily on human and/or mouse genomes. It is now widely accepted that rodents and primates fall within the same supraordinal clade that is often called Euarchontoglires, but is formally named and defined as the crown group Supraprimates (comprising the orders Primates, Dermoptera, Scandentia, Rodentia and Lagomorpha [11]). Supraprimates is recognised as only one of the four main lineages of eutherian mammals, the others being Laurasiatheria (Pholidota, Carnivora, Perissodactyla, Cetartiodactyla, Chiroptera, Eulipotyphla), Xenarthra (Cingulata, Vermilingua, Folivora) and Afrotheria (Proboscidea, Sirenia, Hyracoidea, Tubulidentata, Macroscelidae, plus Afrosoricida = Tenrecomorpha = Chrysochloridae+Tenrecidae) [11]–[15], and not especially close to the root. Significantly, investigations of LINE-1 distribution in species representative of other eutherian clades have demonstrated that Supraprimates display distinct patterns to the others [16], [17].

Here we extend the understanding of LINE-1 using PCR amplification from genomes of a broad range of placental species complemented with Blastn searches of available databases, with emphasis on the two most basal placental clades, Afrotheria and Xenarthra. Using these data we examine how closely a tree of LINE elements follows the generally accepted tree of placental mammals [11]–[15], [18], [19]. Thus, taxon-specific LINE-1 activity is identified. There is strong evidence for autapomorphic groups of LINE-1 active in Afrotheria, Xenarthra and Boreoeutheria, i.e. AfroLINEs, XenaLINEs, and BoreoLINEs respectively.

Finally, LINE and SINE transposition, or site-specific insertion events, are considered rare genomic changes and as such are increasingly used as phylogenetic characters to test other reconstructions [11], [20]. However, the potential for convergent insertions, and the question of how often this occurs, needs to be addressed. The construction of an accurate tree of LINEs across mammals is an essential step in addressing this question.

## Results and Discussion

### Sequence data

**Figure 1:**
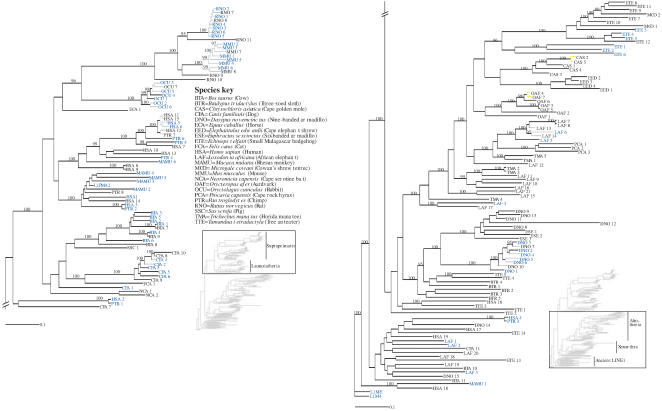
Phylogenetic tree of LINE-1: combined dataset. Bayesian consensus tree generated by a GTR invariant-sites plus Γ model applied to our concatenated dataset (69 long and 109 short sequences; see text for details). Posterior probability values ≥95% are shown. Species in blue reflect sequences that are 1050 bp in length, whereas those in black correspond to the 300 bp sequences. Grey branches indicate sequences with >98% homology in their respective genomes. For species lacking whole genome sequencing projects, that is manatee, hyrax, golden mole, sloth, bat, among others, yellow is used to indicate pairs of sequences with >98% homology. This yields a minimum estimate of the number of copies of potentially recently active L1 in these species. A species key shows the abbreviated names, scientific names and common names. The tree is broken into two sections. The inset shows which part of the tree is displayed.

Since most LINE-1 copies are 5′-truncated and subsequently evolve as pseudogenes [1] they accumulate open reading frame (ORF) terminating, nonsense, and indel mutations. As expected, most of the 52 clones sequenced herein exhibit gaps and are non-active elements. Overall, five aardvark, three golden mole, one elephant and one bat clone displayed an ORF. It is probable that these represent a recent class of transposon not having had sufficient time to acquire mutations in this region. To further determine which lineages appeared recently active, all elements from the extended dataset were BLASTed against their genome of origin, and any that gave full-length hits with homologies higher than 98% have their branches coloured grey in Figure 1. A divergence of 2% is approximately 5 million years at the relatively slow rate that apes (e.g., chimp, human) evolve, or ∼1–3 million years for murid rodents which show the most rapid rates of change (along with tenrec) on our trees. These blast searches can of course be done only in those species for which there are at least whole genome shotgun sequence data (armadillo, elephant, tenrec, human, chimp, macaque, rabbit, mouse, rat, cow, dog). Therefore, to estimate recent activity in the absence of these data (for Cape serotine bat, aardvark, Cape golden mole, Cape elephant shrew, Cape rock hyrax, Cowan's shrew tenrec, Florida manatee, six-banded armadillo, tree anteater, pale-throated three-toed sloth), pairwise distances of closely related LINE-1 were checked, and any pair of sequences that were >98% similar to each other are coloured yellow in the tree (Figure 1). This gives a reasonable estimate of recently active LINE-1 in our dataset.

### The Tree

All trees reveal a similar history, irrespective of the methods used. Figure 1 shows the hierarchical Bayesian consensus tree generated by a GTR invariant-sites plus gamma model. This method with posterior probability (pp) values is useful for a number of reasons. Firstly, with short sequences (and many of these are only 300 bp) the bootstrap approach tends to be very severe on resolving clades. To illustrate this consider, for example, a transversional change that defines a clade without contradiction by other characters. This will receive a bootstrap proportion of ∼67%, which is low. However, if the model identifies this as a substitution pattern that is very unlikely to be due to multiple hits, it should receive a high pp value. Secondly, under the model pp values tend to be conservative [21] yet we need to be cautious since the data do not fit the model so pp values can easily become too extreme as the likelihood function becomes inexact [11]. For other mammalian nuclear genes, pp values seemed to produce few enough strongly misleading results as to retain utility for evaluating branches of trees from short sequences [22]. Finally, and reassuringly, a careful visual inspection of our results shows there are no high (>0.95) pp values that clash with *a priori* expectations.

### Lineage-specific LINEs and systematic implications

**Figure 2:**
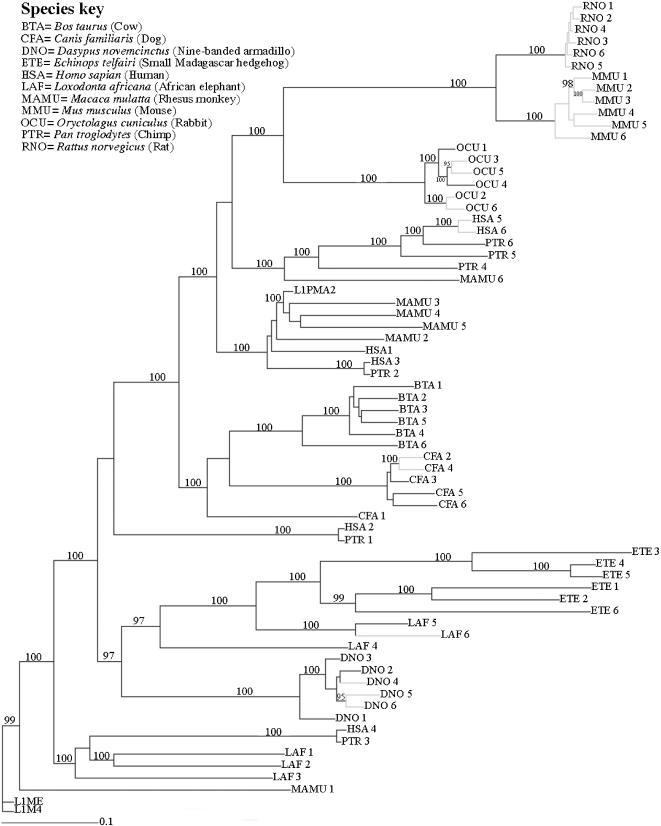
Phylogenetic tree of LINE-1: longer sequences only. Bayesian consensus tree generated by a GTR invariant-sites plus Γ model applied to our long (1050 bp) dataset that included 69 sequences. Posterior probability values ≥95% are shown. Grey branches indicate sequences with >98% homology to other LINE-1 copies in their respective genomes. A species key shows the abbreviated names, scientific names and common names.

Moreover, our two data sets (longer sequences only vs. combined data) retrieved similar topologies (Figure 1 and Figure 2) with the shorter sequences grouping/falling in expected positions around the corresponding longer sequences. Consequently, the following discussion largely focuses on the outcomes reflected in the combined tree (Figure 1). Although we are aware that our investigations represent gene phylogenies, since most sequences are paralogous many clades of LINE-1 elements were, nonetheless, found to reflect closely what is known about species relationships in mammals. This clearly suggests that for many lineages all LINE-1 active at any one time coalesce to a common and not too distant ancestor. Hence these signatures of ancestral but exclusively shared TE activity can reasonably be used as potential synapomorphies, and are thus useful for inferring phylogenetic relationships between species.

#### AfroLINEs, XenaLINEs and BoreoLINEs

Three major clades of L1 appear in our tree corresponding to three main placental lineages. They are monophyletic assemblages of LINE-1 sequences obtained exclusively from: 1) Boreoeutheria represented here by primates (human, chimpanzee and macaque), rodents (rat and mouse) plus lagomorphs (rabbit), i.e. Supraprimates, and Laurasiatheria (pig, cat, bat, horse, cow and dog). 2) Xenarthra represented in our analysis by a monophyletic group of nine-banded and six-banded armadillo L1, with sequences of other xenathrans (sloth and anteater) clustered at their base. 3) Afrotheria represented by elephant, manatee, hyrax, golden mole, elephant shrew, tenrec and aardvark (Figure 1). The support for the monophyly of LINE-1 elements specific to many of these taxa, some of which have high homology to other copies in their respective genomes (and therefore were probably active relatively recently), suggests that there has been continued LINE-1 activity in nearly all these placental lineages. Molecular dating indicates that Afrotheria differentiated from other Placentalia ∼110–95 million years ago (MYA) and subsequently radiated ∼85 MYA [11], [14], [23], [24]. A potentially good geological calibration that agrees with such dates is the separation of Afrotheria and Xenathra by the opening of the South Atlantic about 100 MYA [12].

The activity of a unique family of SINEs in afrotherians (AfroSINES [25]) supports early AfroLINE activity since SINE mobility is reliant on the RTase from transposable elements other than themselves, primarily LINE-1 [8]. Other rare genomic characters supporting Afrotheria include a 9 bp deletion in BRCA1 [26], a 237-246 bp deletion in APOB [27], two chromosomal syntenic associations (HSA1+19q, HSA5+3+21 [28]) and several TE insertions [29].

#### Intra BoreoLINEs

Relationships of active L1 lineages within Boreoeutheria closely follow the expected species relationships. There are monophyletic groups of rat and mouse L1 to which the rabbit elements are basal. Sister to this grouping are primate LINE-1, which include the primate-specific L1PA2 element consensus sequence. Interestingly, there are two clades of primate specific L1, one of which appears to have been recently active with elements that have >98% homology to other copies in the genome. The L1PA2 consensus sequence falls within the clade with no recent activity in human, and therefore most likely represents a LINE-1 lineage that has become extinct in human (Figure 1). Collectively these elements represent supraprimate retrotransposons. Also included in the Boreoeutheria are monophyletic groupings of cow and dog L1 together with the pig, cat and bat L1 that collectively represent laurasiatherian elements. Additionally, we identified three orthologous L1 between human and chimpanzee (as indicated by their identical location and orientation to the same markers in both species). All three pairs share 98–99% homology to each other: HSA_3 and PTR_2 fall within the primate L1; the older HSA_2 and PTR_1 elements fall at the base of Boreoeutheria; and the ancient HSA_4 and PTR_3 fall near the root of the tree with other ancient elements (Figure 1).

### Intra AfroLINEs

Many robust LINE-1 groups were retrieved within Afrotheria. Most hyrax, manatee and elephant LINE-1 sequences were grouped into two clades providing evidence for at least two clade-specific elements, thus strongly consistent with Paenungulata. This suggests that LINE-1 activity and evolution occurred in the ancestral Paenungulate genome subsequent to their differentiation from other afrotherians but before the hyrax/manatee/elephant split [11], [14], [23], [24] which could not be resolved using our LINE-1 investigations.

Equally interesting from the phylogenetic viewpoint is recovery of a large group of LINEs only in taxa of the superorder Afroinsectiphillia (aardvark, tenrecs, golden moles and elephant shrews [11], [14], [28]) and, within it, Afroinsectivora (the former taxa minus aardvark). Indeed all LINEs sampled within these orders coalesce within their respective orders (with the exception of two tenrec sequences obtained from the database). Surprisingly, we do not find support for Afrosoricida (tenrecs plus golden moles). This can be a difficult group to recover using nucleotide sequences [22] and has yet to be confirmed by a suite of conservative characters (although it is consistent with some of the morphology [30]). In our investigation a distinct branching pattern emerged with golden mole closest to elephant shrew (Figure 1).

Within aardvark there is good evidence of recent activity as indicated by our identification of closely related sequences (yellow branches in Figure 1) and the finding of five intact open reading frames (ORFs) in the seven aardvark clones sequenced. This is consistent with fluorescent *in situ* hybridization patterns showing aardvark to be highly enriched with LINE-1 relative to other placental mammals [17] suggesting that this was a relatively recent event which is perhaps ongoing.

### XenaLINEs and the root

Use of the consensus sequences L1M4 and L1ME to root the tree is further justified by a diverse assemblage of apparently very old LINE-1 insertions, shared by many of the main placental lineages, and appearing sister to them. All have seemingly been inactive for a considerable period as suggested by their long terminal lineages plus numerous indels and non-sense mutations (data not shown).

The relationship between the four major placental clades is still hotly debated. There are three competing hypotheses, the Epitheria hypothesis that Xenarthra is sister to all other placentals [20], [31], the Atlantogenata hypothesis that Xenarthra and Afrotheria are sister taxa and sister to all other placentals [32], and the Exafroplacentalia hypothesis that Afrotheria is at the root [11], [14], [22], [33]. Molecular analyses, even using long concatenated sequences, fail to provide consistent statistical support to any of these (compounded by the fact that taxon sampling makes a considerable difference) suggesting the phylogenetic models are breaking down [11], [33]. Recently, however, support for the Epitheria hypothesis was shown by Kriegs *et al* [20] using retroposed elements. We on the other hand show that the L1 sequences from the xenarthran species fall sister to the AfroLINEs (Figure 1) favouring the Atlantogenata hypothesis. In the long dataset tree the AfroLINEs (represented by elephant and tenrec) and XenaLINEs (represented by nine-banded armadillo) association has a pp value of 0.97 (Figure 2). This is also consistent with results by Waddell, Umehara, Griche and Kishino (unpublished) whose analyses of the 17 aligned genomes at the UCSC browser, identify 15 highly conserved indels of 5 bp or greater in favour of Atlantogenata, but only four for Epitheria and three for Exafroplacentalia (a highly significant result by the test in [11]).

There is strong support for two lineages of nine-banded armadillo L1. Members of only one of these clades have homology >98% to other elements in the genome. L1 members of the remaining clade appear to have been inactive for longer with homologies of 92–95% to other L1 copies in the genome. They also cluster with the three L1 isolated from six-banded armadillo and, therefore represent a family of L1 that was active prior to the divergence these two armadillo species (Figure 1).

Assuming our tree is accurate, at least two interpretations of our results can be made depending on which topology of the placental tree is considered correct. First, if either the Exafroplacentalia or Epitheria hypothesis is correct, then a variety of LINEs must have been active just before the three main placental groups split. These remained active after the first branching and by chance a fairly closely related pair of LINE-1 lineages came to dominate the genomes of afrotherians (AfroLINEs) and xenarthrans (XenaLINEs) with all others apparently going extinct. In boreoeutherians, the LINE lineages that came to dominate Afrotheria and Xenarthra went extinct and a third (more distantly related) assemblage of LINE lineages (BoreoLINEs) eventually dominated. The alternative, and *a priori* more likely explanation is that LINEs follow the species tree, and the Atlantogenata hypothesis is correct. Either hypothesis is consistent with what is known presently about LINE evolutionary history and functioning. This includes L1 activity before and during the first branching within placentals [2], [10], plus cycles of competition, extinction and replacement of L1 which is well documented in primates and rodents [34]–[36].

A serious concern in all sequence analyses of placental orders is that the model of sequence evolution assumed is inadequate, and as a consequence there will be systematic errors in the tree that swamp stochastic errors. In order to further assess the potential for these biases we ran a set of transversion only analyses. In general the stochastic error of edges rose but the same basic topology was retrieved. That is, the L1 consensus based root was surrounded by old copies of L1, and there were four main groups of LINEs. Those in taxa from Afrotheria and Xenarthra on one side, and those from taxa within Supraprimates and Laurasiatheria on the other side of the root, thus supporting the Atlantogenata postulate.

Although we are unable to definitely determine which of the hypotheses outlined above is correct, the recently initiated sequencing of complete genomes for a wide variety of mammals including armadillo, elephant, tenrec, rabbit, hedgehog, and guinea pig (see http://www.ensembl.org/index.html) should allow refined testing of the alternatives. Indeed at last count, there were at least 2X draft sequences completed for nearly 30 mammals representing all but four orders - Tubulidentata (aardvark), Sirenia (manatee and dugong), Pholidota (pangolin) and flying lemur (Dermoptera). Such an extensive evaluation will be an important test of how informative a PCR/phylogenetic survey such as this is in determining the history and activity of LINE-1 in a diverse group, and how episodic LINE activity has been in the evolutionary past.

Finally, as the testing of deeper phylogenetic relationships (such as inter-ordinal relationships) moves to include L1 (also SINE and other TE) insertion events [11], [20], the need to sample L1 across many taxa will be a critical test to determine whether particular insertion events can be considered appropriately rare genomic changes. Convergent L1 insertion events will tend to appear paraphyletic within the tree, whereas synapomorphic insertions should be monophyletic. Thus an accurate tree of LINEs for all genomes should go hand in hand with using these characters as phylogenetic markers. Our work represents an important step in this direction.

## Material and Methods

Previously designed general primers [17] were used to amplify approximately 300 bp of ORF 2 (240 bp within the 7^th^ and 8^th^ subdomains of the RT domain plus 60 bp extending into the region directly 3′ to the RT [37]). Such PCR preferentially targets the most numerous intact LINE-1 family members in the genome. PCR products were cloned and a random sample sequenced. Homology of the clones with LINE-1 sequence was systematically verified using the RepeatMasker program (http://repeatmasker.genome.washington.edu).

Two phylogenetic data matrices were prepared. The first included 66 LINE-1 sequences of 1050 bp from 11 eutherian species (six sequences from each species) with genome sequencing projects available from www.ensembl.org ( Supplementary table 1) plus three consensus sequences (L1PMA2, L1M4 and L1ME [10]). Our second matrix included these 69 sequences (1050 bp), plus 52 additional ∼300 bp sequences we obtained by PCR, and 57 partial sequences from Genbank. This second matrix comprised 1050 characters and 178 sequences representing a total of 22 placental species in 13 orders (Supplementary table 1). The shorter 300 bp sequences are homologous to the 3′ end of the 1050 bp sequences. Sequences were aligned using T-coffee v1.35 [38] then refined manually. Nucleotide sites that clearly appeared to be post-transposition insertions (e.g., insertions present in only one sequence that also cause a major frameshift) were removed from the data set.

A variety of methods were used to explore the data. These included parsimony, distance based trees, and maximum likelihood (using PAUP* [39]), plus a “hierarchical Bayesian” method implemented using (MC)^3^ chains (MrBayes 3.0 [40]). The hierarchical Bayesian (or marginal likelihood) trees, in particular, tended to best reflect biological expectations by recovering well-established clades suggesting fewer errors in reconstruction. General transition matrices, such as the HKY [41], or general time reversible GTR models with site rate variability following an invariant sites (p_inv_) plus gamma (Γ) distribution [42]–[44], were used for model-based methods. The Γ distribution was approximated with four discrete rate classes of equal size. Five chains (four hot, one cold) plus a random starting tree was used for each (MC)^3^ run. Chains were run to at least 4 million steps with sampling of trees every 50 steps. Plateaus (supposed convergences) in likelihood tended to appear by ∼200,000 steps, but all trees prior to 500,000 steps were discarded in order to be conservative. All other settings were left at their defaults. Each model was run at least twice and both topology and posterior probability (pp) values of edges were checked for conformity between runs.

To further test deeper portions of the tree, and in particular possible misrooting, transversion only invariant-sites plus gamma models were used [42]. Since the average instantaneous transversion rate is approximately four times less than that of transitions, this should strongly reduce the extent to which multiple hits confound tree estimation. These were implemented by converting all As in the data into Gs and all Cs into Ts. PAUP* and MrBayes then default to transversion models. Note that, in the case of MrBayes, the Dirichlet priors remained those for four states, but these allowed the frequencies of A and C to go to much less than zero, closely approximating a transversion only model.

Serious issues with the rooting of placentals using sequence data are: (1) Breakdown of the fit of data to any currently used phylogenetic model, (2) the long distance to the marsupial outgroup [26], and (3) long unbranched ingroup sequence [29]. In the present investigation the root of the tree was identified *a priori* as a L1M4 consensus sequence and a L1ME (ancient L1M4 subfamily) consensus sequence which was postulated as predating the earliest splits among living placental mammals [10]. Using consensus sequences of L1 common to all placentals offers a root much closer than marsupials, while L1 elements themselves break up long branches of the species tree such as the aardvark lineage (the only living species in Tubulidentata). These two effects together offer considerable robustness against break-down of the model [42]. To further increase robusteness, we ran transversion-only models to explore the root.

## Supporting Information

Table S1 Species and common names of sequences used in this study. The sequence names are as they appear on the phylogeny of Figure 1. The location of the sequence is given as a position on either a chromosome, scaffold or clone, along with its accession number. Sequences generated in this study have an accession number but no strand location as these are not part of a genome assembly.
(0.06 MB XLS)
